# Determination by near infrared microscopy of the nitrogen and carbon content of tomato (*Solanum lycopersicum* L.) leaf powder

**DOI:** 10.1038/srep33183

**Published:** 2016-09-16

**Authors:** Gauthier Lequeue, Xavier Draye, Vincent Baeten

**Affiliations:** 1 Université catholique de Louvain, Earth and Life Institute - Agronomy (ELI-A), de Serres Building, Croix du Sud 2, L7.05.11, 1348 Louvain-la-Neuve, Belgium; 2 Walloon Agricultural Research Centre. Valorisation of Agricultural Products Department, Food and Feed quality Unit, Henseval Building, Chaussée de Namur 24, 5030 Gembloux, Belgium

## Abstract

Near infrared microscopy (NIRM) has been developed as a rapid technique to predict the chemical composition of foods, reduce analytical costs and time and ease sample preparation. In this study, NIRM has been evaluated as an alternative to classical chemical analysis to determine the nitrogen and carbon content of small samples of tomato (*Solanum lycopersicum* L.) leaf powder. Near infrared spectra were obtained by NIRM for independent leaf samples collected on 216 plants grown under six different levels of nitrogen. From these, 30 calibration and 30 validation samples covering the spectral range of the whole set were selected and their nitrogen and carbon contents were determined by a reference method. The calibration model obtained for nitrogen content proved to be excellent, with a coefficient of determination in calibration (R^2^_c_) higher than 0.9 and a ratio of performance to deviation (RPD_c_) higher than 3. Statistical indicators of prediction using the validation set were also very high (R^2^_p_ values > 0.90). However, the calibration model obtained for carbon content was much less satisfactory (R^2^_c_ < 0.50). NIRM appears as a promising and suitable tool for a rapid, non-destructive and reliable determination of nitrogen content of tiny samples of tomato leaf powder.

For four decades, the food sector has adopted near-infrared (NIR) based techniques for the quantitative control of raw materials and final products. These techniques offer the possibility to investigate simultaneously physical, biological and nutritional features of complex matrices, require small sample amounts and simple small preparation, and involve small analytical costs and times^1^,^2^,^3^,^4^,^5^,^6^. The number of NIR or mid-infrared (MIR) applications is continuously increasing. The high sample throughput of these techniques have been used to predict several qualitative and quantitative features of fruits, vegetables, grains, oils, tea and other agricultural products, as substitute or complement of conventional destructive methods^1^,^4^,^7^,^8^,^9^,^10^,^11^,^12^,^13^. In particular, they have proven as fast and routinely applicable alternatives to conventional methods of quantification of the total protein content, e.g. the Kjeldahl method (total organic nitrogen content), the Dumas method (total nitrogen content) and spectroscopy (infrared absorbance of proteins)^3^,^5^,^14^,^15^.

NIR spectra of food products include mainly absorption bands characteristic of O–H, C–H, N-H, S-H and C-C groups. These bands are the result of the interaction between photons and matter. These interactions occurring in the NIR region of the electromagnetic spectrum induce vibration transitions in the second, third or higher excited states or overtones, as well as combinations derived from fundamental vibrations that occur in the MIR region^5^,^16^,^17^. NIR spectra quality is impacted by various factors, such as the physical state of the product (solid or liquid), temperature of the sample, granulometry (e.g. powder or non-concrete products), homogeneity and presence of impurities^6^.

In plant science, NIR-based methods offer the possibility to identify and quantify primary and secondary metabolites without preliminary physical separation. Barbin *et al.*^4^ and Krähmer *et al.*^5^ have recently assigned the most characteristic NIR bands of some primary (e.g. carbohydrates, lipids, proteins) and secondary (e.g. phenolic substances, terpenoids, alkaloids) metabolites^4^,^5^. One of the main limitations in plant research remains the need to harvest sufficient amounts of material, typically for experiments with Arabidopsis or for phenotyping experiment aiming at mapping metabolites in an organ. The combination of near-infrared spectrometry (NIRS) with microscopy appears to be a viable solution to address this challenge. With this technique, the NIR spectra of a sample area as small as 1 μm^2^ can be collected non-destructively^6^.

NIR microscopy (NIRM) is a relatively novel technique that enables the spectral analysis of individual particles. NIRM was first used in feed analysis to detect forbidden animal protein in compound^18^,^19^. Many studies have demonstrated the value of NIRM for producing high-quality spectra from small particles (<500 μm)^20^,^21^,^22^,^23^,^24^,^25^,^26^,^27^,^28^. Because NIRM results can be shared easily in networks of laboratories^28^, the technique has been validated at European level^27^.

This pioneering study evaluates the value of NIRM to predict the nitrogen and carbon contents (e.g., main primary plant metabolites) in tiny samples (<40 mg) of tomato leaf powder.

## Material and Methods

### Plant material and growth conditions

The tomato (*Solanum lycopersicum* L.) variety Ailsa Craig was used in this study. The experiment was conducted in Louvain-la-Neuve, from 23 July 2013 to 12 September 2013. Seeds were surface-sterilized by soaking in a 5% (v/v) sodium hypochlorite solution for 15 min and rinced three times with deionized water. Seeds were germinated in a loam substrate incubated in a growth chamber (24 °C/22 °C; 80% RH; 16 h photoperiod; 150 μmol.m^−2^.s^−1^ PAR). Ten days after sowing, unrooted cuttings were washed with deionized water and transferred individually in 1.45 L pots filled with a mix of perlite and vermiculite (50/50).

After seven days of rooting in the growth chamber, the cuttings were transferred in a greenhouse for seven days acclimation period. A data-logger (TinyTag Ultra, model TGU-1500, INTAB Benelux, Netherlands) was used to record climate data during the experiment (T_mean_ 26.5/18.2 °C day/night, (max. 34.8/27.9 °C day/night, min. 13.1/12.8 °C day/night) and RH_mean_ 52.8/69.0% day/night (max. 93.5/96.4% day/night, min. 27.8/41.6% day/night). The photoperiod was set at 16 h and the solar radiation was supplemented with Philips HPLR lamps (400 W) providing 40 μmol m^−2^ s^−1^ at the canopy level. During these periods, plants were watered three times per week using a modified Hoagland solution^29^ with a nitrogen concentration of 13 mmol.l^−1^ (Table 1).

The set of plants was then splitted into six groups of 12 plants which were exposed to one of six nitrogen concentrations (13.0; 6.50; 3.25; 1.63; 0.81; 0.41 mmol.l^−1^) (Table 1). They were watered three times per week with a volume of 100 ml solution from the top of the pot.

From the start of the treatment application, three plants were harvested weekly in each treatment. The shoot and root parts were dried by oven-drying at 65 °C until constant weight. Finally, dry aerial parts were crushed with a sample mill (CT 193 Cyclotec™, Foss, Hillerød, Denmark) to obtain a powder (with a dry matter weight between 0.01 and 2.41 g).

The complete experiment was performed in three simultaneous full repetitions, generating 216 samples in total.

### NIR microscopy

The near infrared analyses were performed using a completely automated Fourier Transform-IR imaging Microscope (Hyperion 3000, Bruker Optics, Ettlingen, Germany). Data were recorded in the range from 9.000 to 4.000 cm^−1^ with a spectral resolution of 8 cm^−1^.

All spectra were collected with 32 co-added scans. Vibrational spectroscopy was performed directly on crushed shoot powder. For each sample, 10 spectra were collected at different spatial location of the samples spread on an aluminum plate with 96 wells containing the sample allocated in 2 or 5 wells, based upon the dry weight available. After the analysis of the sample, the file including the 10 spectra collected was opened in the Opus 6 (Bruker Gmbh, Germany) to verify the presence of the characteristic NIR bands and the absence of noisy spectra.

**Figure 1:**
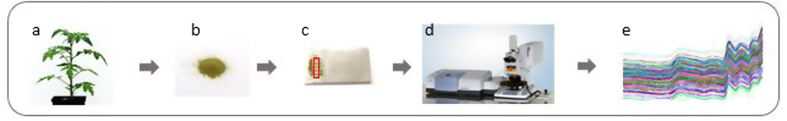
Workflow of the NIRM analysis performed. First step is the reduction of leaf (**a**) to powder (**b**) and after this powder is spread into the 96 well plate (**c**). The plate is then presented to the microscope (**d**) and 10 NIR spectra are collected at different locations (**e**).

One of the samples was analyzed several times during the three days of measurement to determine the value of inter-day reproducibility. The subsequent chemometric evaluation has exclusively been based on the average spectra on all samples. Figure 1 shows the workflow of the analysis process by NIRM.

Thirty samples of the total set (216) have been used to construct the model (calibration set) and thirty others to validate the model (validation set). The calibration and validation sets analyzed were selected to cover the full range of NIR spectral variation.

### Reference analysis

The nitrogen (N-value in %) and carbon (C-value in %) content of each sample of the calibration and validation sets were determined by combustion according to the Dumas method using 5 mg of powder. The analysis was carried out on an elemental analyzer (Flash EA 1112 series, Thermo Finnigan, San Jose, CA, USA). The time interval separating the measurements of these two sets was three months during which the samples were stored in hermetic pots conserved in dark room. The calibration curves for the elemental analyzer were determined by using atropine standard to different known concentration of carbon and nitrogen contents and routinely checked using this standard. Six samples from the calibration sets were measured in duplicate (the second analysis was performed at the same time as the analysis of the validation set). This allowed to check the stability and reproducibility of the reference method and estimate the error of the elemental analyzer.

### Statistical analysis

Multivariate chemometric analysis was performed using the Unscrambler^®^ X software version 10.3 (Camo Inc., Oslo, Norway) and in accordance with the considerations formulated by Dardenne (2010), summarized below^30^. The standard error of the reference method (SEL, also called reproducibility) was calculated as the mean of the standard deviations of the difference between the duplicates of six samples of the calibration set that were measured at a three-month interval. The raw NIR spectra were preprocessed using Savitsky-Golay algorithm to compute smoothed (noise reduction), first derivative (offset and bias removal) spectra. The smoothing window did not eliminate any important feature of the spectra. Accordingly, all the relevant chemical informations were retained for modeling. The NIRM model was built with the following workflow: (1) establishing a NIRM calibration model for target compositions and then optimizing this model; (2) using validation sets to verify the accuracy and repeatability of this model and (3) finally, to improve the accuracy of the prediction, the calibration and validation sets were combined to elaborate the final NIRM model.

The development of the NIRM calibration model linking NIRM data (X) with chemical data (Y) was performed using Partial Least Squares (PLS) regression and a cross-validation procedure^31^,^32^. The number of latent variables was selected by the software. A cross-validation with the leave-one-out method was performed by dividing into 2 segments the data matrix, containing 15 or 30 samples, respectively, for the calibration and final NIRM models.

The accuracy of each calibration (for the calibration and final NIRM models) was evaluated based on the coefficients of determination (R^2^) for predicted versus measured compositions in cross-validation and prediction, and the ratio of prediction to deviation (RPD)^31^. The RPD showed the ratio between the standard deviation (SD) of data set to standard error of calibration (SEC) or standard error of cross-validation (SEC_cv_). The SEC, which expresses the accuracy of NIR results corrected for the mean difference between NIR and reference methods (bias), was calculated by the equation (1)^33^:


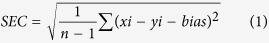


where x_*i*_ − *y*_*i*_ is the difference between results obtained by the NIRM method (x_*i*_) and reference method (*y*_*i*_) on sample *i*, and *n* is the total number of independent samples in the test. Bias is the difference between the average of results obtained by the NIRM method (x_*i*_) and reference method (*y*_*i*_) on sample^33^.

In the validation step of the calibration model, the determination coefficient of prediction (R^2^_P_), the standard error of prediction (SEP) and the root mean square of prediction errors (RMSE_P_) values was used to evaluate the accuracy of the model^30^. The RMSE_P_ was calculated from the difference between NIRM and reference results by the following equation (2)^33^:


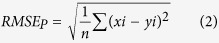


where *x*_*i*_ − *y*_*i*_ is the difference between results obtained by the NIRM method (*x*_*i*_) and reference method (*y*_*i*_) on sample *i*, and *n* is the total number of independent samples in the test. The residual standard deviation (RSD) was represented the errors after bias and slope correction or the errors along the calculated single regression line (with a loss of two degrees of freedom)^34^.

The R^2^ was obtained for the models according to the following equation (3)^30^:


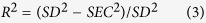


For the validation step of the calibration model, SEC was replaced by SEP.

The accuracy of the predictions for the models was considered as excellent when R^2^ ≥ 0.91, good when 0.90 ≥ R^2^ ≥ 0.82, moderately successful when 0.81 ≥ R^2^ ≥ 0.66, and unsuccessful when R^2^ ≤ 0.65^31^. In this study, five levels of prediction accuracy were considered for the RPD value of the calibration and the final NIRM models. The accuracy of the intermediate NIRM and final NIRM calibration model was considered unreliable for a RPD < 1.5, a RPD between 1.5 and 2.0 allowed to distinguish the high and low values, good for a RPD between 2.0 and 2.5, a value between 2.5 and 3 allowed to an approximate quantitative predictions and excellent for a RPD > 3^31^. The RPD was directly linked to R^2^ (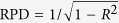) and the RPD was anyway more discriminant than R^2^ especially when high R^2^ is close to 1^30^. The interpretation of the prediction accuracies based on the R^2^ and RPD values was useful to compare the prediction accuracy of different models considered.

## Results and Discussion

### Spectra description

**Figure 2:**
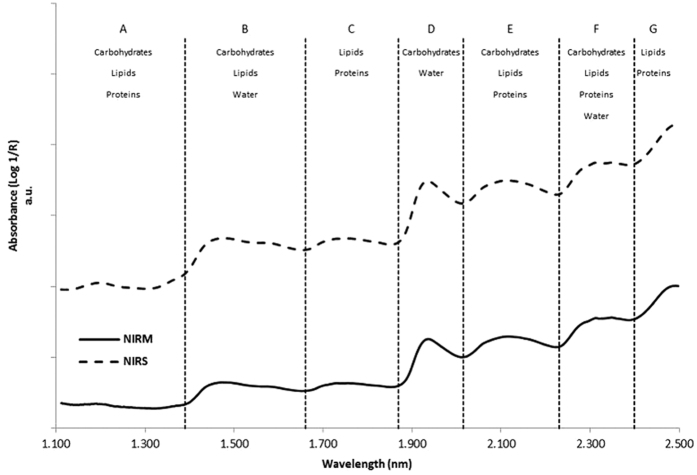
Comparison between the near-infrared spectra of one of our samples analyzed by NIRM instrument (continuous line; Hyperion, Bruker Optics, Germany) and by NIRS classical instrument (dotted line; XDS, Foss, Denmark) with the attribution of the main infrared bands (**A**–**G**). For sake of clarity, spectra were shifted on the Y (Absorbance) axis.

A total of 2.160 raw spectra were obtained from an acquisitions at 10 different spatial locations on each of the 216 samples. The chemometric evaluation has been based on the average spectrum of each sample. Most of the variation between locations and samples was observed in the absorbance from 1.887 to 2.439 nm (5.300 to 4.100 cm^−1^) range. Figure 2 illustrates the similarities between near-infrared spectra for one of our samples analyzed by NIRM and by classical NIRS instrument in the range between 1.100 to 2.500 nm (9.091 to 4.000 cm^−1^) with a spectral resolution of 8 cm^−1^. As mentioned earlier in the study of Yang *et al.*^26^, the spectrum characteristics obtained by NIRM correspond to those of NIRS. Main of the absorption bands are observed in the 1.660–2.500 nm (6.024 to 4.000 cm^−1^) range which is mainly related to carbohydrates, lipids and crude protein^26^.

The main features of the absorption bands of the two spectra were clearly visible on the Fig. 2. No differences in the bands position or in the shape were observed between the spectra acquired NIRS and NIRM technologies. The spectra could be decomposed into 7 main sections from low to high wavelengths (Fig. 2). The first one was characteristic of the second overtone of symmetric and anti-symmetric C-H stretch vibration (-CH, -CH_2_ and -CH_3_ groups) absorption (A) from 1.100 to 1.390 nm (9.091 to 7.194 cm^−1^). These absorption bands are related to the content in carbohydrates, lipids and proteins^4^,^5^,^16^,^17^,^35^. The second region was characteristic of the first overtone of the O-H vibration bands and the intermolecular H-bridges of water absorption (B) from 1.390 to 1.660 nm (7.194 to 6.024 cm^−1^). There was also an overlap with the combination of two stretches and one deformation of C-H bonds producing this broader NIR absorption band, related to the content in carbohydrates and lipids^4^,^5^,^16^,^17^,^35^. The third region was characteristic of the first overtone of symmetric and anti-symmetric C-H stretch vibration (-CH_2_ and -CH_3_ groups) absorption (C) at 1.660 and 1.870 nm (6.024 and 5.348 cm^−1^), respectively. These absorption bands are related to the content in lipids and proteins^4^,^5^,^16^,^17^,^35^. The next region includes absorption bands characteristic of the first overtone (D) from 1.870 to 2.015 nm (5.348 to 4.963 cm^−1^). These absorption bands are mainly related to the content in carbohydrates^4^,^16^,^17^,^35^. The broader absorption around 1.934 nm (5170 cm^−1^) was also due to an overlap with combinations derived from the vibration of O-H bands characteristic of absorption by water and fundamental vibrations of ester bands (C = O) that occur in the MIR region^4^,^5^,^17^. The fifth region was characteristic for the absorption of C-H, N-H and C=O bonds present in carbohydrates, lipids and proteins (E) from 2.015 to 2.230 nm (4.963 to 4.484 cm^−1^), corresponding to the combination of C-H stretching and bending modes of methyl (-CH_3_) and methylene (-CH_2_) functional groups^4^,^5^,^16^,^17^,^35^. The next region is characteristic of the C-H combination bands for carbohydrates, lipids and proteins absorption (F) from 2.230 to 2.360 nm (4.484 to 4.237 cm^−1^)^4^,^5^,^16^,^17^,^35^. The last region is characteristic of the C-H combination bands for lipids and proteins absorption (G) from 2.360 to 2.500 nm (4.237 to 4.000 cm^−1^)^4^,^5^,^16^,^17^,^35^.

### Reference analysis

The minimum, maximum, mean, and standard deviation (SD) of the nitrogen and carbon content (N and C-value in %) in the calibration and validation sets are shown in Table 2.

The value range for the nitrogen and carbon content in the calibration and validation sets were similar which means that the calibration and validations sets can be used to establish, test and verify the accuracy of the NIRM model. Reference values of calibration and validation sets were showed in Supplementary Table S1.

### NIRM calibration and validation

NIRM calibration models were developed for the nitrogen (models 1, 2 and 3) and carbon (model 4) content determination using the 30 samples of the calibration set and the differences between the models are summarized in Table 3.

In accordance with the recommendations of Dardenne (2010), Table 4 summarizes the characteristics of the models constructed^30^. The calibration step highlights the presence of 3 outliers for the N-value. For the nitrogen content calibration, the best compromise for the number of terms used to derive the calibration was 5 or 3, respectively for models constructed without (e.g., raw data) or with pre-treatments (e.g., smooth and derivative). The low difference between the standard error of calibration (SEC) and the standard error of cross-validation (SEC_cv_) for the N-content models was indicated a sufficient number of samples for the calibration. In this study, the determination coefficient of calibration (R^2^_c_) of the first model (model 1) was 0.86 and the SEC_cv_ was 0.31 (Table 4). The calibration models with pre-treatments, have R^2^_c_ values were 0.90 and 0.98, respectively good for model 2 and excellent for model 3. The determination coefficient of cross validation (R^2^_cv_) values were closely aligned with the R^2^_c_ values for both calibration models (Table 4), albeit typically a little weaker than R^2^_c_. SEC_cv_ for the nitrogen content determination were 0.27 and 0.14, respectively for model 2 and model 3.

In this study, the ratio of prediction to deviation of calibration (RPD_c_) for the nitrogen content calibration were 3.16 and 7.07 respectively for the calibration model 2 and model 3. These results correspond to excellent models^31^.

For the carbon content calibration, the best model built has a R^2^_c_ value of 0.20 (model 4) and a RPD_c_ value of 0.88 (Table 4). In accordance with Saeys *et al.*^31^, this results indicate that it was not possible to build a successful calibration^31^. Trial to build a successful calibration has been also done using a databases made on the 60 samples used for the calibration and the validation sets. The best R^2^_c_ value was 0.30 and RPD_c_ value was 0.84. To conclude, a good model to predict C-value content in tomato leaves powder could not be achieved.

**Figure 3:**
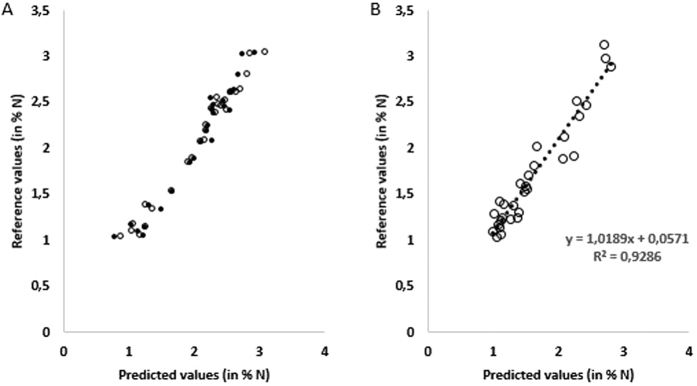
(A) Plot of % N in the *Solanum lycopersicum* L. samples analyzed in the calibration stage. Results of the reference values vs NIRM prediction (Model 3) are plotted. NIRM calibration (o) and cross-validation (**·**) results are displayed. **(B)** Plot of % N in the *Solanum lycopersicum* L. samples analyzed in the validation stage. Results of the reference values vs NIRM prediction (Model 3) are plotted.

Model 3 (pretreatment and outliers exclusion) was selected on the basis of the SEC, R^2^_c_ and RPD_c_ values and was tested on the validation set. Figure 3A displays calibration and cross-validation results (the reference values *versus* NIRM predicted values) of Model 3 for determination of N-value in %.

The performance of NIRM model 3 was tested on the 30 independent samples of the validation set (Table 5). The determination coefficient of prediction (R^2^_p_) obtained on the validation set was 0.93 for the nitrogen content determination (Table 5). This result of the validation step indicates that the accuracy of the predictions of NIRM model 3 was excellent (R^2^ ≥ 0.91).

The standard error of prediction (SEP) obtained on the independent validation set was 0.16 for the nitrogen content determination. The SEP of NIRM model is expected to be equal or superior to the standard error of reference method (SEL, also called reproducibility). In this NIRM model (Table 5), the SEP value (0.16) was just three times higher than the SEL values (0.05). The SEP value demonstrates the possibility to predict accurately the N content. The root mean square error of prediction (RMSE_P_) obtained using Partial Least Square (PLS) after pre-processing optimization was 0.18 for total nitrogen content. Figure 3B presents the reference values versus NIRM predicted values obtained for N-content (in %) for the samples of the validation set.

In order to improve the accuracy of the prediction, the data of the calibration and validation sets (60 samples) were combined to elaborate the models 5 and 6, respectively, with and without outliers (Table 5). A cross-validation procedure was used to evaluate the quality of these models. Four outliers were highlighted for the final N-value set of samples. The narrow gap between SEC and SEC_cv_ for models 5 and 6 indicated that the number of samples included in the study is adequate.

The SEC values obtained for models 5 and 6 were 0.18 and 0.11 respectively, about two and three times higher than the SEL (0.05) of the reference method (Table 6). The increase in the number of samples achieved by combining the two sets therefore improved the performances of the model (Models 3 and 6 have, respectively, a SEP of 0.16 (Table 4) and a SEC of 0.11 (Table 6)). The coefficient of determinations (R^2^_c_) obtained for models 5 and 6 were 0.91 and 0.97 respectively, indicating that the performances of the two models were excellent. Finally, models 5 and 6 yield RPD_c_ of 3.33 and 5.77 respectively, which correspond to excellent prediction models. Model 6 (pretreatment and outliers exclusion) was finally selected on the basis of the SEC, R^2^c and RPDc values to make predictions. Predictions results of the model 6 were showed in Supplementary Table S2.

## Conclusions

Our study demonstrates the feasibility of accurately estimating the N content of very small tomato leaf samples using the NIRM technique. The main benefits of this technique compare to conventional methods (e.g. the Kjeldahl method, the Dumas method and NIRS) lays essentially in the simple sample preparation procedure, involve small analytical costs and times and in the small amount of tissue that is required. This innovation should ease (i) the establishment of N profiling among different organs of the same plant, (ii) the dynamic monitoring of N content in time for a given plant and (iii) the development of high throughput methods of N quantification in studies involving large numbers of genotypes. Conditional on further validation, the method may also proved very useful for small plants such as *Arabidopsis thaliana* where large amounts of plant material often requires the pooling of several plants. In a N profiling strategy, the methodology may also be used to produce local observations within a leaf, especially in the study of defense mechanisms against leaf diseases.

One may expect the NIRM methodology to be used for predicting other physical, chemical and biological properties and for embracing different aspects of the plant phenotype or phenotypic responses to various factors. The potential of the NIRM method to detect plant stress due to abiotic factors (e.g., nutrients, salinity) and to determine the chemical and physical properties in several plants tissues and samples (e.g., whole plants, fruits, grains, leaves) has been demonstrated already^1^,^4^,^7^,^8^,^9^,^10^,^11^,^12^,^13^. The ongoing technical improvements of NIRM will offer new perspectives and solutions for a fast, reliable, environmentally-friendly testing and simultaneously quantification of physical, chemical and biological plant properties.

## Additional Information

**How to cite this article**: Lequeue, G. *et al.* Determination by near infrared microscopy of the nitrogen and carbon content of tomato (*Solanum lycopersicum* L.) leaf powder. *Sci. Rep.* **6**, 33183; doi: 10.1038/srep33183 (2016).

## Supplementary Material

Supplementary Information

## Figures and Tables

**Table 1:** The principal chemical compounds (in mmol.l^−1^) with the different electrical conductivity (EC in dS/M) and pH used in nitrogen solutions (1.3; 6.50; 3.25; 1.63; 0.81; 0.41 mmol.l^−1^) achieve from a modified Hoagland solution.

Elements	Solutions					
[Total nitrogen] (in mmol.l^−1^)	13	6.50	3.25	1.63	0.81	0.41
NO3^−^ (in mmol.l^−1^)	12.00	6.00	3.00	1.50	0.75	0.38
NH4^+^ (in mmol.l^−1^)	1.00	0.50	0.25	0.13	0.06	0.03
SO4^2−^ (in mmol.l^−1^)	1.00	3.75	5.13	5.82	6.16	6.33
H2PO4^−^ (in mmol.l^−1^)	2.01					
K^+^ (in mmol.l^−1^)	6.01					
Ca^2+^(in mmol.l^−1^)	3.50					
Mg^2+^ (in mmol.l^−1^)	1.00					
pH	5.6					
EC (dS/m)	1.60	1.51	1.53	1.51	1.51	1.50

**Table 2:** Characteristics of the calibration and validation sets.

	Calibration set		Validation set	
**Units**	% N	% C	% N	% C
**N**	30	30	30	30
**Min**	1.03	31.33	1.03	35.44
**Mean**	2.09	37.27	1.71	38.79
**Max**	3.08	40.00	3.12	41.86
**SD**	0.64	1.50	0.60	1.11

**Units**: percentage of N or C; **N**: number of samples; **Mean**: average; **SD**: standard deviation; **Min**: minimum; **Max**: maximum.

**Table 3:** Differences between calibration models.

Model	Dependent	Pre-treatement	Outlier exclusion
1	N	None	None
2	N	Yes	None
3	N	Yes	Yes
4	C	Yes	None

**Model**: model number constructed (1, 2, 3 and 4); **Dependent**: Nitrogen (N) or Carbon (c); **Pre-treatment**: with or without data pre-treatment; **Outliers exclusion**: with or without excluded values.

**Table 4:** Characteristic of the NIRM models constructed.

Parameters	N-value	N-value	N-value	C-value
**Model**	1	2	3	4
**Units**	% N	% N	% N	% C
**SEL – reproducibility**	0.05	0.05	0.05	1.19
**N**	30	30	30	30
	0	0	3	0
**Min**	1.03	1.03	1.03	31.33
	2.09	2.09	2.07	37.27
	3.08	3.08	3.04	40.00
**SD**	0.64	0.64	0.635	1.50
**SEC**	0.22	0.20	0.09	1.32
**R^2^C**	0.86	0.90	0.98	0.20
**SECCV**	0.31	0.27	0.14	1.45
**R^2^_CV_**	0.78	0.81	0.95	0
**Reproducibility - between days**	0.32	0.32	0.32	3.52
**Number of terms**	5	3	3	3
**RPD_C_**	2.67	3.16	7.07	0.88
**RPD_CV_**	2.13	2.29	4.47	0.97
**Segments (CV)**	2	2	2	2
**WL Range/Resolution (nm)**	1111–2500/2	1111–2500/2	1111–2500/2	1111–2500/2
**Pre-treatement(s)**	NONE	SG-D1 (15, 2)	SG-D1 (15, 2)	SG-D1 (15, 2)
	PLS	PLS	PLS	PLS

**Model**: model number constructed on all sample or without outliers; **Units** : percentage of N or C; **SEL – reproducibility**: standard error of the reference method; **N**: number of samples; **Outliers**: number of values excluded; **Min**: minimum; **Mean**: average; **Max**: maximum; **SD**: standard deviation for N or C-values of the calibration set; **SEC**: standard error of calibration; **R^2^**_**C**_: determination coefficient of calibration; **SEC**_**CV**_: standard error of cross-validation; **R^2^**_**CV**_: determination coefficient of cross-validation; **Reproducibility between days**: variability of the measurements between days; **Number of terms**: number of terms in the equation; **RPD**_**C**_: ratio of prediction to deviation of calibration; **RPD**_**CV**_: ratio of prediction to deviation of cross-validation; **Segments (CV)**: segments in the cross-validation procedure; **WL range/step (nm)**: range of wavelengths explored and resolution in nm; **Pre-treatment(s)**: data pre-treatment apply on raw spectra; **Reg. Method**: regression method used.

**Table 5:** Statistics of the NIRM model 3 validation for the nitrogen determination.

Parameters	N-value
**Units**	% N
**N**	30
**Outliers**	0
**Min**	1.03
**Mean**	1.71
**Max**	3.12
**SD**	0.60
**R^2^_P_**	0.93
**RMSE_P_**	0.18
**SEP**	0.16
**RSD**	0.20
**Reproducibility - between days**	0.32
**Bias**	−0.09
**Intercept**	0.06
**Slope**	0.91

**Units** : percentage of N; **N**: number of samples; **Outliers**: number of values excluded; **Min**: minimum; **Mean**: average; **Max**: maximum; **SD**: standard deviation for N-values of the validation set; **SEC**: standard error of calibration; **R^2^**_**P**_: determination coefficient of prediction; **RMSE**_**P**_: root mean square errors of prediction; **SEP**: standard error of prediction; **RSD**: the residual standard deviation; **Reproducibility between days**: variability of the measurements between days; **Bias**: deviation of the regression line; **Intercept**: regression constant; **Slope**: coefficient de regression.

**Table 6:** Characteristics and statistics of the final NIRM model.

Parameters	N-value	N-value
**Model**	5	6
**Units**	% N	% N
**SEL – reproducibility**	0.05	0.05
**N**	60	60
**Outliers**	0	4
**Min**	1.03	1.03
**Mean**	1.90	1.86
**Max**	3.12	3.04
**SD**	0.64	0.62
**SEC**	0.18	0.11
**R^2^_C_**	0.91	0.97
**SEC_CV_**	0.22	0.14
**R^2^_CV_**	0.89	0.95
**Reproducibility - between days**	0.32	0.32
**Number of terms**	3	3
**RPD_c_**	3.33	5.77
**RPD_cv_**	3.02	4.47
**Segments (CV)**	2	2
**WL Range/Resolution (nm)**	1111-2500/2	1111-2500/2
**Pre-treatement(s)**	SG-D1 (15, 2)	SG-D1 (15, 2)
**Reg. Method**	PLS	PLS

**Model**: model number constructed on all sample or without outliers; **Units** : percentage of N; **SEL – reproducibility**: standard error of the reference method; **N**: number of samples; **Outliers**: number of values excluded; **Min**: minimum; **Mean**: average; **Max**: maximum; **SD**: standard deviation for N-values of the calibration and validation sets combined to elaborate the final model; **SEC**: standard error of calibration; **R^2^**_**C**_: determination coefficient of calibration; **SEC**_**CV**_: standard error of cross-validation; **R^2^**_**CV**_: determination coefficient of cross-validation; **Reproducibility between days**: variability of the measurements between days; **Number of terms**: number of terms in the equation; **RPD**_**C**_: ratio of prediction to deviation of calibration; **RPD**_**CV**_: ratio of prediction to deviation of cross-validation; **Segments (CV)**: segments in the cross-validation procedure; **WL range/step (nm)**: range of wavelengths explored and resolution in nm; **Pre-treatment(s)**: data pre-treatment apply on raw spectra; **Reg. Method**: regression method used.
