# Supplementary material for: Determination by near infrared microscopy of the nitrogen and carbon content of tomato (Solanum lycopersicum L.) leaf powder
Source: Sci Rep. 2016 Sep 16;6:33183. doi: 10.1038/srep33183 (PMC5025744; doi:10.1038/srep33183)
Supplement: Supplementary Information [file srep33183-s1.pdf]

Supplementary Data for:

Determination by near infrared microscopy of the nitrogen and carbon content of tomato (*Solanum lycopersicum* L.) leaf powder.

Gauthier Lequeue[1], Xavier Draye[1] & Vincent Baeten[2,*]

[1] Université catholique de Louvain, Earth and Life Institute - Agronomy (ELI-A), de Serres Building, Croix du Sud 2, L7.05.11, 1348 Louvain-la-Neuve, Belgium.

[2] Walloon Agricultural Research Centre. Valorisation of Agricultural Products Department, Food and Feed quality Unit, Henseval Building, Chaussée de Namur 24, 5030 Gembloux, Belgium.

*corresponding author: v.baeten@cra.wallonie.be

Supplementary Table S1. Reference analysis values (N and C-content in %) of the calibration and validation sets based on experimental parameters (treatments [1.3; 6.50; 3.25; 1.63; 0.81; 0.41 mmol.l^{-1}], plant numbers [1 to 12], date of harvest [22/08/2013, 28/08/2013, 5/09/2013 or 12/09/2013] and repetition [1, 2 or 3]).

Treatment parameters				Reference analysis	
**[Total nitrogen] (in mmol.l^{-1})**	**Plant number**	**Harvest date**	**Repetition**	**N-value (in %)**	**C-value (in %)**
13.00	5	5/09/2013	1	2.35	39.28
13.00	10	22/08/2013	1	2.54	39.30
6.50	6	5/09/2013	1	1.71	39.31
6.50	9	29/08/2013	1	1.88	36.60
6.50	11	22/08/2013	1	3.04	38.70
3.25	1	12/09/2013	1	1.40	38.74
3.25	5	5/09/2013	1	1.49	37.05
3.25	6	5/09/2013	1	1.56	39.11
1.63	1	12/09/2013	1	1.15	38.56
1.63	4	5/09/2013	1	1.43	37.97
1.63	6	5/09/2013	1	1.38	37.43
0.81	1	12/09/2013	1	1.07	39.16
0.81	7	29/08/2013	1	2.08	36.39
0.81	11	22/08/2013	1	2.52	41.36
0.41	3	12/09/2013	1	1.10	38.74
0.41	4	5/09/2013	1	1.31	38.74
0.41	12	22/08/2013	1	2.18	36.38
13.00	1	12/09/2013	2	2.43	37.61
13.00	2	12/09/2013	2	2.18	38.20
13.00	3	12/09/2013	2	1.91	35.44
13.00	10	22/08/2013	2	3.08	40.00
6.50	1	12/09/2013	2	1.34	37.32
6.50	4	5/09/2013	2	1.53	38.66
6.50	12	22/08/2013	2	2.51	37.36
3.25	3	12/09/2013	2	1.24	38.45
3.25	4	5/09/2013	2	1.59	38.16
3.25	9	29/08/2013	2	2.47	38.50
1.63	3	12/09/2013	2	1.23	38.73
1.63	7	29/08/2013	2	1.89	38.39
0.81	2	12/09/2013	2	1.18	38.23
0.81	9	29/08/2013	2	2.02	38.64
0.41	2	12/09/2013	2	1.09	36.73
0.41	7	29/08/2013	2	1.81	38.92
0.41	8	29/08/2013	2	2.07	38.02
0.41	11	22/08/2013	2	2.61	38.96
0.41	12	22/08/2013	2	2.80	37.73

Supplementary Table S1 (continued). Reference analysis values (N and C-content in %) of the calibration and validation sets based on experimental parameters (treatments [1.3; 6.50; 3.25; 1.63; 0.81; 0.41 mmol.l^{-1}], plant numbers [1 to 12], date of harvest [22/08/2013, 28/08/2013, 5/09/2013 or 12/09/2013] and repetition [1, 2 or 3]).

Treatment parameters				Reference analysis	
**[Total nitrogen] (in mmol.l^{-1})**	**Plant number**	**Harvest date**	**Repetition**	**N-value (in %)**	**C-value (in %)**
13.00	2	12/09/2013	3	2.38	37.42
13.00	6	5/09/2013	3	3.02	37.78
13.00	7	29/08/2013	3	2.63	37.51
13.00	11	22/08/2013	3	2.98	41.86
6.50	5	5/09/2013	3	1.52	37.36
6.50	10	22/08/2013	3	2.89	39.78
3.25	5	5/09/2013	3	1.61	38.48
3.25	7	29/08/2013	3	2.12	38.78
3.25	10	22/08/2013	3	2.41	36.97
3.25	11	22/08/2013	3	2.46	38.82
1.63	1	12/09/2013	3	1.38	38.42
1.63	3	12/09/2013	3	2.25	31.33
1.63	4	5/09/2013	3	3.12	40.51
0.81	1	12/09/2013	3	1.18	37.17
0.81	2	12/09/2013	3	1.06	36.85
0.81	5	5/09/2013	3	1.24	38.50
0.81	6	5/09/2013	3	1.21	38.47
0.81	10	22/08/2013	3	2.60	35.96
0.41	1	12/09/2013	3	1.03	38.06
0.41	2	12/09/2013	3	1.03	37.30
0.41	4	5/09/2013	3	1.29	37.72
0.41	5	5/09/2013	3	1.14	35.53
0.41	7	29/08/2013	3	1.85	36.33
0.41	11	22/08/2013	3	2.47	37.85

Supplementary Table S2. Predicted Nitrogen values (in %) which have been obtained by the final NIRM model (model 6) based on experimental parameters (treatments [1.3; 6.50; 3.25; 1.63; 0.81; 0.41 mmol.l^{-1}], plant numbers [1 to 12], date of harvest [22/08/2013, 28/08/2013, 5/09/2013 or 12/09/2013] and repetition [1, 2 or 3]).

Treatment parameters			Predicted values of Nitrogen (in %)		
[Total nitrogen] (in mmol.l^{-1})	Plant number	Harvest date	Repetition 1	Repetition 2	Repetition 3
13.00	1	12/09/2013	2.70	2.29	2.10
13.00	2	12/09/2013	2.72	2.11	2.43
13.00	3	12/09/2013	2.06	2.30	2.21
13.00	4	5/09/2013	2.38	2.92	2.37
13.00	5	5/09/2013	2.40	2.05	2.18
13.00	6	5/09/2013	2.54	2.33	2.91
13.00	7	29/08/2013	2.32	2.39	2.72
13.00	8	29/08/2013	2.77	2.76	3.36
13.00	9	29/08/2013	2.86	2.42	2.75
13.00	10	22/08/2013	2.37	2.45	2.28
13.00	11	22/08/2013	2.49	2.75	2.90
13.00	12	22/08/2013	2.96	2.40	2.57
6.50	1	12/09/2013	1.54	1.40	1.47
6.50	2	12/09/2013	1.52	1.47	1.56
6.50	3	12/09/2013	1.81	1.45	1.57
6.50	4	5/09/2013	1.43	1.52	1.68
6.50	5	5/09/2013	1.43	1.72	1.72
6.50	6	5/09/2013	1.71	1.71	1.51
6.50	7	29/08/2013	2.54	2.51	1.97
6.50	8	29/08/2013	2.34	2.27	2.18
6.50	9	29/08/2013	1.96	1.69	2.87
6.50	10	22/08/2013	2.92	2.71	2.91
6.50	11	22/08/2013	3.06	2.30	2.33
6.50	12	22/08/2013	2.40	2.46	2.69
3.25	1	12/09/2013	1.20	1.46	1.54
3.25	2	12/09/2013	1.03	1.65	1.83
3.25	3	12/09/2013	1.11	1.44	1.67
3.25	4	5/09/2013	1.42	1.56	1.34
3.25	5	5/09/2013	1.03	1.51	1.53
3.25	6	5/09/2013	1.60	1.70	1.42
3.25	7	29/08/2013	2.21	2.01	2.17
3.25	8	29/08/2013	2.81	2.11	2.56
3.25	9	29/08/2013	2.18	2.50	2.53
3.25	10	22/08/2013	2.30	2.57	2.52
3.25	11	22/08/2013	2.56	2.60	2.38
3.25	12	22/08/2013	2.76	2.64	2.81

Supplementary Table S2 (continued)**.** Predicted Nitrogen values (in %) which have been obtained by the final NIRM model (model 6) based on experimental parameters (treatments [1.3; 6.50; 3.25; 1.63; 0.81; 0.41 mmol.l^{-1}], plant numbers [1 to 12], date of harvest [22/08/2013, 28/08/2013, 5/09/2013 or 12/09/2013] and repetition [1, 2 or 3])**.**

**Treatment parameters**			**Predicted values of Nitrogen (in %)**		
**[Total nitrogen] (in mmol.l^{-1})**	**Plant number**	**Harvest date**	**Repetition 1**	**Repetition 2**	**Repetition 3**
1.63	1	12/09/2013	1.15	1.18	1.35
1.63	2	12/09/2013	1.22	1.24	2.45
1.63	3	12/09/2013	1.30	1.23	2.15
1.63	4	5/09/2013	1.18	1.39	2.75
1.63	5	5/09/2013	1.17	1.18	1.18
1.63	6	5/09/2013	1.37	1.19	1.20
1.63	7	29/08/2013	1.85	2.09	1.49
1.63	8	29/08/2013	2.46	2.12	1.41
1.63	9	29/08/2013	2.27	2.25	1.29
1.63	10	22/08/2013	2.88	2.13	1.94
1.63	11	22/08/2013	2.57	2.32	2.25
1.63	12	22/08/2013	2.55	2.38	2.59
0.81	1	12/09/2013	1.17	1.17	1.08
0.81	2	12/09/2013	1.28	1.10	1.20
0.81	3	12/09/2013	1.12	1.09	1.12
0.81	4	5/09/2013	1.26	1.52	1.47
0.81	5	5/09/2013	1.61	1.41	1.20
0.81	6	5/09/2013	1.56	1.62	1.22
0.81	7	29/08/2013	2.18	2.36	2.02
0.81	8	29/08/2013	2.45	2.18	2.29
0.81	9	29/08/2013	2.04	1.87	2.07
0.81	10	22/08/2013	2.76	2.20	2.61
0.81	11	22/08/2013	2.43	2.49	2.29
0.81	12	22/08/2013	2.60	2.68	2.51
0.41	1	12/09/2013	1.18	1.07	1.15
0.41	2	12/09/2013	1.23	1.13	0.98
0.41	3	12/09/2013	1.08	1.08	1.18
0.41	4	5/09/2013	1.37	1.37	1.17
0.41	5	5/09/2013	1.21	1.33	1.30
0.41	6	5/09/2013	1.97	1.23	1.32
0.41	7	29/08/2013	1.97	1.74	1.85
0.41	8	29/08/2013	2.55	2.21	2.33
0.41	9	29/08/2013	2.23	2.01	2.10
0.41	10	22/08/2013	2.32	3.04	2.55
0.41	11	22/08/2013	2.34	2.53	2.50
0.41	12	22/08/2013	2.94	2.71	2.77
